# Lessons from patient and parent involvement (P&PI) in a quality improvement program in cystic fibrosis care in France

**DOI:** 10.1186/s13023-017-0751-9

**Published:** 2018-02-08

**Authors:** Dominique Pougheon Bertrand, Guy Minguet, Rémi Gagnayre, Pierre Lombrail

**Affiliations:** 1 LEPS EA3412, Sorbonne Paris Cité University, Bobigny, France; 2 Mines-Nantes School, Nantes, France

**Keywords:** Cystic fibrosis, Quality improvement program, Patient involvement, Patient engagement

## Abstract

**Background:**

Quality Improvement Programs (QIP) in cystic fibrosis (CF) care have emerged as strategies to reduce variability of care and of patient outcomes among centres facilitating the implementation of Best Practices in all centres. The US CF Foundation developed a Learning and Leadership Collaborative program which was transposed in France in 2011. Patient and parent involvement (P&PI) on the local quality teams (QTs) is one dimension of this complex intervention. The conditions and effects of this involvement needed to be evaluated.

**Methods:**

In all settings, patients and parents were recruited by their centre care team. They were trained to QI method and tools and contributed their own expertise to improve the process of care. This involvement has been analyzed in the frame of the whole process evaluation. Observations and interviews conducted during the course of the first training year explored the motivations of the patients and parents to participate and the vision of the health care teams. A research study was carried out after three years with the patients/parents and the professionals to assess the French QIP’s effectiveness using a questionnaire to report their opinions on various components of the program, including their experience of P&PI. Responses were analyzed in view of identifying consensus and dissensus between the two groups.

**Results:**

At the introduction of the program, P&PI was an opportunity for healthcare providers to reflect on their conceptions of these individuals both as patients and as healthcare system users. Curiosity about the teams’ functioning, the various center organizations and outcomes led patients to overcome their initial barriers to participation. Seventy-six people including 12 patients/parents from the 14 pilot centres responded to the questionnaire after 3 years. Consensus between professionals and patients/parents was high on most items characterizing the performance of the QIP, QT effectiveness and QT functioning. Patients, parents and professionals agreed on the main characteristics of care such as an optimized organization, multidisciplinary care and patient-centredness. Regarding the use of patient electronic records, the use of care guidelines or the organization of support in the patient community, responses were not consensual amongst patients/parents and a source of dissensus between the two groups. All agreed that the French QIP created good conditions for their involvement. In the end, both groups agreed that it was difficult to attribute the paternity of some changes specifically to any member in the team.

**Discussion:**

Perspectives such as an educational framework to develop the skills and behaviors of professionals engaged in collaborative practice with patients and families and large patient experience surveys could be used to capture patients’ experience of care in the improvement work.

**Conclusion:**

Success factors for patient/parent long-term involvement in QIPs have been identified. Answers to questions raised by the stakeholders about the feasibility, efficiency and usefulness of P&PI in this CF QIP could be given but new questions arose about the sustainability of continuous quality improvement over time.

## Background

Patient involvement in quality of care improvement is discussed in various ways depending on the perspective and the point of care delivery.

Quality of care in hospital settings was defined by the US Institute of Medicine in 2001 as clinical effectiveness, safety and patient centredness [1]. Clinical effectiveness is generally viewed as too technical to accommodate patient contributions and the usefulness of patient surveys in assessing medical quality of care remain debatable [2]. However, it is widely accepted that patients may make significant contributions to non-clinical aspects of care [3]. Many opportunities have been identified for patients to contribute to the safety of the care they receive at the hospital [4]. Moreover, reporting of safety information on medical errors and adverse events through patient interviews or surveys may also aid in identifying failures in every stage of the care process, from diagnosis to medication or clinical services [5]. Therefore, patients are recognized as being capable of contributing substantially to safety in the care by identifying care factors that potentially lead to harm or helping to learn from an incident to avoid it in the future [6]. Beyond matters of safety, the involvement of patients or their representatives in the organization of hospital care is usually associated with activities related to planning services, designing processes or assessing quality management. Groene and Sunol proposed a conceptual framework for patient involvement in quality management comprising 5 stages: criteria development, process design, quality committees, improvement projects and discussion of quality improvement project results [7]. Their literature review and a cross-sectional survey at hospitals in the DUQuE project [8] reported experiences of patients involvement across these stages [9]: 1) on guideline development to address the needs of chronically ill patients as well as aspects of continuity of care and integration of service; 2) in assessing care preferences and designing process through surveys, focus groups and observations; 3) in regular formal meetings to ensure quality and safety; 4) in establishing a partnership with the QI team to plan and deliver a QI intervention in a series of plan-do-study-act (PDSA) cycles; 5) more rarely in discussing quality improvement project results.

The history of cystic fibrosis (CF) care has been one of continuous improvement, led by the worldwide combined efforts of patient organizations, researchers and clinical teams. Therapeutic advances associated with the implementation of CF specialized care centres have brought about a dramatic increase in life expectancy and quality of life for people with CF. In the new century, Quality Improvement Programs (QIP) have emerged as new strategies to reduce variability in care as well as in patient outcomes across centres facilitating implementation of Best Practices in all centres. In this rare disease, QI is driven by comparisons of patient outcomes between national patient registries at national and centre levels [10]. Since the 2000s, the US CF Foundation and the Dartmouth Institute have developed a CF Learning and Leadership Collaborative (LLC) program to accelerate the improvement of CF care across the US centres [11]. French clinicians and patient organization representatives gained awareness of this quality improvement program during international CF meetings and considered that its transposition to the French CF network was an opportunity to accelerate the improvement of care with the current therapeutics.

Since newborn screening became generalized in France in 2002, the French CF care network has been organized into specialized CF centres (CFCs). In the frame of the second French National Plan for Rare Diseases two centres of expertise were designated in order to develop French national action plans. The US CF QIP was then transposed to France by the Nantes-Roscoff centre of expertise, and the PHARE-M *(Programme Hospitalier d’Amélioration des Résultats et de l’Expertise en Mucoviscidose – A hospital-based program for improvement of results and expertise in cystic fibrosis care)* program was launched in September 2011 through a pilot phase involving 14 centres volunteer to test and adapt the method to the French CF care organization (Table 1) [12]. This QI approach is innovative in France as it installs a quality improvement dynamics and culture among the health care teams focusing on disease specific care practices and patient health outcomes improvement [13] when most QI interventions are framed by the French National Health Authority certification process. PHARE-M intends to involve patients and parents on a long-term collaboration with their care teams to take into account their experience and preferences along the successive PDSA cycles for the redesign of the care process at their centre. This is part of the innovation of this QI approach in France which needed to be evaluated.

**Table 1:** Number of Patients at the CFC engaged in PHARE-M by year

CF Program	Year PHARE-M	# Patients Data 2014	Pilot PHASE 2011–2013
PEDIATRIC			
Angers	2013	122	122
Bordeaux	2016	148	
Clermont-Fd	2013	103	103
Créteil	2015	109	
Dunkerque	2015	71	
Grenoble	2013	122	122
Lille	2015	181	
Lyon	2012	290	290
Nancy	2016	113	
Nantes	2012	104	104
Paris R Debré	2012	168	168
Rennes	2013	131	131
Roscoff	2012	75	75
Tours	2016	116	
Vannes-Lorient	2013	81	81
Versailles	2012	65	65
ADULT			
Lyon	2012	313	313
Nantes	2013	203	203
Rennes	2013	101	101
Montpellier	2015	197	
Reims	2012	131	131
Roscoff	2013	75	75
TOTAL Patients in PHARE-M Group		3019	2084
% Patients recorded in Registry		47%	33%

The objective of this article is to report and reflect on patient and parent involvement at the 14 centres engaged in the pilot phase of the PHARE-M program from the perspective of the patients and parents and from the perspective of the professionals on the quality teams. By illustrating Groene’s conceptual framework regarding *the partnership between patients and the QI team to plan and deliver a QI intervention in a series of plan-do-study-act (PDSA) cycles*, we intend to contribute to the field with our experience of patient/parent involvement in a learning and leadership quality improvement program within a rare disease network in France.

## Methods

Some aspects of P&PI were particularly questioned from the point of view of both the patients/parents and the professionals: how did they perceive the conditions in place to allow the participation of patients and parents in the program? How did the quality team’s professionals perceive this participation and what were the feelings of the participating patients and parents? Is the quality of care appreciated in the same way by patients and professionals after three years of joint work? How effective were the quality teams perceived in organizing the QI work and mastering the QI method and tools to which they had been trained? How effective was the participation of all members in the discussions and in decision-making? In the end, was the contribution of patients / parents perceptible in the quality improvement work and on the results on the process of care?

We present successively the conditions set up for patient and parent involvement in the PHARE-M program then how their involvement has been analyzed, first in the evaluation of the transposition process of the US QIP to France, then in the assessment of the program’s effectiveness after three years [14].

### Setting: Patient and parent involvement in the PHARE-M

The PHARE-M is a one year training program that follows a step by step curriculum known as the Dartmouth Microsystem Improvement Ramp [15] consisting of multiple steps described in this OJRD supplement [12].

**Fig. 1:**
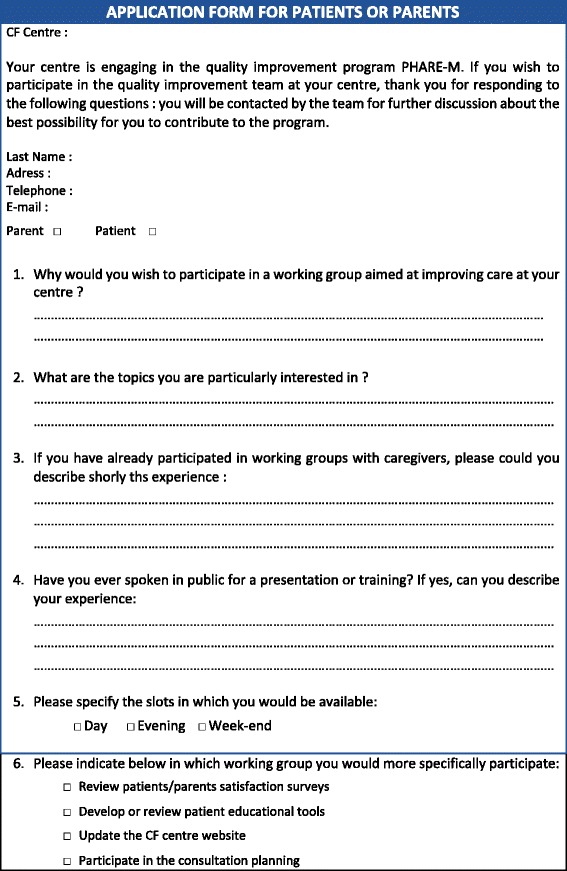
Criteria for Patient or Parent application form

The quality team (QT) formed at each CFC involves 4 to 5 professionals from the multidisciplinary team and is led by a physician. The recruitment of a parent (pediatric program) or a patient (adult program) in the quality team is a prerequisite to engage in PHARE-M. It has been operated by the physician leader following a recruitment procedure including a list of criteria on an application form (Fig. 1). Volunteer patients and parents were recruited after information given on the QI program and on the importance of their involvement to improve care at their centre. The consent form specifies that neither their participation nor their withdrawal would have any impact on their own care or their child’s care and that their participation in the QT can cease at any time they wish. One « correspondent » professional is in charge for liaising with the patient or parent to regularly share information, answer their questions and solve practical issues. When recruited, patients and parents are enlisted in the PHARE-M training sessions as QT members. They exercise the method with their team during the face-to-face-meetings. Patient outcomes as well as key process indicators are transparently shared with them, those regarding their centre as well as those regarding the other centres involved in the training session. Patients or parents are also invited to participate in the PHARE-M web conferences every 4 to 6 weeks. Their travel fees are reimbursed by the national patient organization. They are invited at the local QT meetings which are generally hold every 2 to 3 weeks. If they can’t attend these meetings, they are updated on the work done by their correspondent on the QT. All personal health information from patients included in redesigned care processes are anonymized before being discussed at any QT meetings attended by the patient or parent. Ethical rules are established in relation to the information shared at the meetings. When a patient or parent group is active at the centre, rules are defined for communication with the group.

### P&PI analysis as part of the transposition process evaluation (year 1)

An evaluation was requested by the leader of the Centre of Expertise as part of the transposition process of the US CF QI program to France [12]. It was conducted by a sociologist from Mines Nantes School on the PHARE-M pilot session in order to investigate requirements for a successful national roll-out of the PHARE-M, identify the possible technical or cultural barriers and propose possible adjustments to the program to adapt it to the French context.

The assessment included becoming familiar with PHARE-M documents. The assessor participated as an observer in five web meetings (January 17, January 31, March 6, April 23, June 14, 2012) and two Face-to-Face meetings (Marseille (March 29) and Nantes (May 22)) and a ccordination meeting of the national team (January 11, 2012). The assessor conducted interviews:

• Onsite visits of 2 centers and focus groups with the teams;

• Individual telephone interviews with all the CRCM Referents of the pilot phase (7), other volunteer members of the steering teams (16), members of the project and support team (4);

• Interview with the Instructor from The Dartmouth Institute for Clinical Practice and Clinical Practice (Marseille on March 28th).

All interviews and focus groups were recorded and fully transcribed. The data was managed (coding, categorization), processed (analysis, validity) and interpreted according to the standard thematic content analysis protocol (Miles & Huberman, 2003 [16]). This was followed by manual grouping and counting within a framework for analysis with the following dimensions: process applicability (terminology, formalization, tools, remote coordination); patients and parents involvement (roles, time spent, obstacles); French national and regional coordination (roles, nature of support, mechanisms for incorporation); process adoption (perceived benefits and costs, working atmosphere, engagement, acquisitions); and effects (operation, working practices, cooperation with partners). *Only results on the dimension regarding patient and parent involvement during the pilot PHARE-M training year are reported in this article.*

### P&PI analysis as part of PHARE-M effectiveness assessment after 3 years

A research project was drawn up by the Centre of Expertise of Nantes-Roscoff to analyze the performance of the PHARE-M program after three years (2015) at the 14 CF centres involved in the pilot phase of the program. This research project was funded by the French ministry of Health (Decision of the Call for project PRePS – Dec 2012). The aims and protocol of the broader project from which the results are drawn are described in the OJRD supplement [17]. In brief, the protocol combines a quasi-experimental evaluation of the effectiveness of the program on patient outcomes evolution over three years with a process evaluation [18]. Following a realistic approach, the latter was designed to understand what works, for whom and under which circumstances (context) [19]. To understand which dimensions of the context were critical for the effectiveness of the programme, a questionnaire was designed assembling existing validated tools when they existed and developing new tools when necessary [17].The questionnaire was composed of 7 chapters covering the various aspects of the organization of care and the PHARE-M effectiveness at the centres: quality of the care process, organizational culture, patient centredness, leadership, mastering of the QI process and tools, quality team functioning and patient/parent involvement.

- Variables

The items in five chapters were based on existing instruments validated in previous research [20, 21]. The items characterizing the chapter about quality of care were developed for this research following the 5 dimensions of the Chronic Care Model [22]. The items of the questionnaire analyzing patient and parent involvement were developed according to the framework proposed by Carman [23] and adapted by Pomey []: 1) patient and parent information/activation 2) patient and parent empowerment and 3) patient and parent contribution to the QI work (Table 2).

- Data collection

**Table 2:** Engagement of the patients/parents on the quality team (QT)

Information and activation of the patients/parents	1. The patients and parents are educated regularly (annually or more often) by the team about general subjects concerning cystic fibrosis care and research.
	2. The patients and parents are rather familiar with general cystic fibrosis information: research, progress made, and Registry data.
	3. The CFC team has educated the patients and parents about the PHARE-M’s importance and aim.
	4. A good relationship between the patient or parent recruited and the team is indispensable for the patient or parent to participate in the PHARE-M.
	5. The patient or parent recruited is well informed of the challenges (10 commitments) of management quality.
	6. The presence of a patient or parent on the steering team is a given and an asset.
	7. The place of a parent or patient is not on a quality team, because he or she does not have enough training or education.
	8. The place of a parent or patient is not on a quality team, because he or she already has too many personal problems to manage.
	9. The patient or parent recruited possesses the qualities to become a member of the steering team.
	10. The patient or parent recruited must have developed coping skills (see therapeutic education standard: knowing how to manage emotions and stress; solving problems, making decisions, and making choices; knowing how to communicate and being adept in relationships with others; and knowing how to put oneself in the place of others).
Empowerment of patients/parents to allow them participate in the QT	1. The participation of a patient or parent depends on the systematic reimbursement of his or her travel expenses.
	2. The participation of a patient or parent should be facilitated by the reimbursement of other expenses: child-care, lost working hours, etc.
	3. The participating patient or parent does not represent all patients.
	4. The patient or parent was selected by the team based on a list of specific criteria (cultural level, capacity to communicate, availability, etc.).
	5. The patient or parent is motivated to improve management for all.
	6. The patient or parent is also motivated to improve his or her own management by participating in the program.
	7. It is important to communicate with the other patients or parents concerning the role of the patient or parent on the steering team.
	8. It would be necessary to include several patients or parents to ensure that more different points of view are represented.
	9. The patient or parent must be knowledgeable about the disease and its management beyond the requirements of his or her own care.
	10. The patient or parent must be knowledgeable about the general functioning of the hospital.
	11. The patient or parent must know how to communicate with the professionals by taking a step back and drawing general lessons from his or her own experience.
Capacity for effective contribution of the patients/parent	1. The PHARE-M national organization created good conditions for incorporation of the patient or parent.
	2. The participation of a patient or parent on the team at French national training and information meetings (four French national face-to-face “EPE” meetings) is indispensable.
	3. The patient or parent participated and contributed as much as the professionals during the French national “EPE” meetings.
	4. The patient or parent’s regular participation at quality team meetings at the CFC is indispensable.
	5. The patient or parent participates in and contributes significantly to the work of the steering team.
	6. The patient or parent’s ideas and proposals are generally taken into account by the steering team.
	7. The atmosphere of work of the steering team meeting is better and more productive when the patient or parent is present.
	8. The pace of work is slower when the patient or parent is present at the steering team meeting.
	9. Certain decisions made by the steering team are inspired by the patient/parent.
	10. The process of incorporation and participation of the patient or parent should be reviewed and improved for the continuation of the PHARE-M.

The questionnaire was self-administrated during 14 on site investigations conducted by a clinical research assistant. Every professional in the 14 centres, including professionals belonging to the quality teams and the patients and parents involved. The respondents had no opportunity to discuss their answers amongst themselves. Each topic is covered by a list of assertions requiring a response on a 5 degrees Lickert scale from « completely agree », to « fully disagree » with a neutral response « don’t know/no opinion ».

- Data analysis

The responses were managed using SAS and XL and were analyzed, according to the purpose, grouping different categories of respondents: professionals in the quality teams, patients and parents. During restitutions to the centre teams, reports by centres were produced to share the results and discuss new improvement goals for the care process.

To analyse the responses from the point of view of the patients/parents and the professionals, the analysis of the responses on all items of the questionnaire was made for the two groups of respondents: the patient/parent group (*N* = 12) and the professional group in the quality teams pooled for all teams and all disciplines (*N* = 64). We first identified the items that achieved a « strong consensus » in the patient/parent group considering unanimous or nearly unanimous responses (unanimity less one vote or unanimity less two votes; >80%) as either positive (grouping « agree » and « completely agree »), negative (grouping « disagree » and « fully disagree ») or neutral (« don’t know » or « no opinion »). We then identified the items that achieved a strong consensus in the professional group (> 80% responses with either positive, negative or neutral answers). We define dissensus or consensus between the patient/parent group and the professional group using Fisher’s exact test [24] (Results available on request).

The results highlighted the following categories: 1) items achieving a consensual position between the two groups of respondents (consensual positions were found always in the same sense in the 2 groups, positive (+), negative (−) or neutral (N)); 2) items achieving consensual position in the patient group only; 3) items achieving consensus in the professional group only; and 4) items achieving no consensus (NC) in either of the two groups (Table 3).

**Table 3:** Presentation of consensus/dissensus between the Patient/Parent and the Professional groups

Items category	Consensus amongst P&P	No consensus (NC) amongst P&P
Consensus amongst Professionals	1) (+,+) or (−,-) or (N,N)	3) (NC,+) or (NC,-) or (NC,N)
No consensus (NC) amongst Professionals	2) (+,NC) or (−,NC) or (N,NC)	4) (NC,NC)

Due to the small sample of patients and parents (*N* = 12) and their affiliation to 12 different centres, variations in their responses regarding local culture, organization, leadership and the performance of the QIP achieving no consensus are mainly to be attributed to “centre effects”. We did not set out to compare the responses of the patient/parent to the responses of the professionals by center.

## Results

### Results from the observations and interviews conducted after year 1

Only the opinions and concerns regarding the participation of parents and patients involved in the QTs after Year 1 are summarized in Table 4. The following themes emerged:

- The place of the patient/parent in the healthcare system

**Table 4:** Opinions, concerns, and illustrative quotes regarding P&PI

Opinion	Concern	Quote
Patients/parents involvement in the Quality Teams		
The place of the patient/parent in the health system	This involvement upset assigned places, led to readjustments and reinterpretations, and highlighted resilient P&P profiles.	Physician: “Certain physicians are not ready to accept that there is a patient at the medical staff meeting, or a meeting like the ones that we have, who gets up and disagrees, who bursts in as a consultant who gives his or her opinion.” Parent1: “I can see that parents who are often negative or react badly to certain situations are parents who are suffering. Sometimes I feel that I stand out from other people, because I am very optimistic by nature and I have a fighting spirit. This may be why I always go a little bit beyond.”
Reason for participation by Parents	They affirmed contributing their testimonial on their experience and sticking to merely conveying their feelings and day-to-day experiences.	Parent2: “I do not aim to teach anyone in a medical setting their profession — one day a physician told me that I was not going to teach him his profession. In participating, I contribute my testimonial as a parent, and that is all. More than anything else, I want to contribute my positive energy and fighting spirit.” Parent3: “My motivation in participating in the meeting with the pediatric team is being able to give my position as a parent. So I am going to tell them my feelings regarding some of their actions. Sometimes, when I tell them my feelings, they are surprised and tell me that they had not seen things in that way.”
Reasons for Patient involvement from their perspective	Wariness: patients were waried of a medicalized world. Consent and curiosity: to get to know a setting, to better get to know the teams that they visited as their care providers. Engagement under tension between: *on the one hand*, the desire to understand, be curious, gain autonomy and confidence, and remove obstacles, and, *on the other hand*, the difficulty of pushing oneself to talk in front of others about one’s experiences with an invasive disease that one would like to keep at a distance.	Patient1: “The idea of meeting with the physicians stressed me out a bit. I wondered what I was going to do, what I should say, how it was going to go.” Patient2: “The differences that there could be between different hospitals were quite astonishing. For example, the outcomes in FEV1% were quite impressive compared to the outcomes we had. You saw that there were distinctly better figures than what we had, indeed... So that was a bit striking to me. It was also interesting to see how other hospitals functioned and provided care, and what could be done to improve quality for patients, basically.” Patient3: “I gave my opinion on the feasibility of things. It is all well and good to say, ‘We have to do X drainages, X treatments, X thingies, etc.,’ but in the end, there is real life which is different from hospital life.”
Projection of healthcare providers on patients in QT	The presence of a patient on the team questions healthcare providers’ professional ideas and desire. It is tempting for healthcare providers to authorize themselves to have a particular conception of patients and parents and then to talk about them, about what they believe to be their experience, in the name of healthcare providers’ experience and in-depth knowledge of the person — his or her journey and record.	Nurse: “It would also be necessary to critique healthcare providers. Healthcare providers need to create the patient’s needs. That is what they do and they are proud of it. Nevertheless, it assumes having a patient who is completely ideal, compliant, etc. Such a patient does not exist. We do not know such a patient. We have never seen one before. These healthcare providers’ pushes always make me very afraid, because I do not lose sight of the fact that they are about the ideal of healthcare providers.” Nurse: “Sometimes, saying that people do not know their disease suits us well in the end, because we will be able to have an effect on them, to explain and re-explain to them. These people understand very well and live with their disease on a day-to-day basis better than us. I do not think that we have the slightest idea of what they are really going through. They know very well what this disease is about, that the final outcome is death. When these patients relax their efforts, we should respect this and not necessarily go and add things.”

Patient and parent involvement disrupted assigned places, led to readjustments and reinterpretations, and highlighted resilient patient and parent profiles.

- Reasons and barriers expressed by parents for participating

They stressed contributing their testimonial on their experience and sticking to merely conveying their feelings and day-to-day experiences. They were careful not to appear to teach professionals their profession.

- Reasons and barriers expressed by patients for participating:
  - Wariness/caution towards the care team and the medical world.
  - Consent and curiosity to get to know a CF setting, to better get to know the teams that they visited as their care providers.
  - Engagement under tension between on one hand, the desire to understand, be curious, gain autonomy and confidence, remove obstacles, and, on the other hand, the difficulty of pushing oneself to talk in front of others about one’s experiences with the care of a disease that one would like to keep at a distance.
- Healthcare providers’ vision of patients/parents involved in the quality teams:

Their vision of patients/parents was confronted with real patients and parents. The presence of a patient on the team called into question healthcare providers’ preconceived notions and desire. Some healthcare providers recognized that they granted themselves the authority to have a particular vision of patients and parents and to talk about them, about what they believe to be patients’ experience and feelings, given their in-depth knowledge of the « ill human being ». The presence and intervention of a real patient or parent in the quality team challenged their representation and some raised the question of the representativeness/validity of the speech of the patient or parent involved.

The patient or parent participation on the QTs and their presence at the PHARE-M Face-to-Face training sessions as well as at many local meetings was perceived as an opportunity for the healthcare providers to reflect on their conceptions of the patients/parents as both patients and healthcare system users. Curiosity about the teams’ functioning and comparison between the various center organizations and their outcomes led patients to overcome their initial barriers and grant their consent to participate.

### Results from the PHARE-M effectiveness assessment after year 3

Over the 3 years, three parents stopped their participation. One parent wanted to stop because of health worsening of her child and was replaced by another parent who happened to be a quality engineer in pharmaceuticals. One pediatric CFC stopped the program when the physician leader retired. The 3rd CFC chose to work with the parent group (as historically) and collect feedback on change actions at annual patient group meeting.

During on site investigations 140 people from the 14 CFCs completed the questionnaire, either as QT participants or as multidisciplinary team members outside the QTs. The QT respondents totaled 76 people (54% of all respondents): 12 patients and parents (6 patients and 6 parents) and 64 professionals, including 56 healthcare providers and 8 non-healthcare providers (quality engineers and others). Two CFCs were unable to contact the patient or parent to ask them to complete the questionnaire. Forty-six (82%) professionals in the QTs belonged to the CF multidisciplinary “core” team (physician, nurse, physiotherapist). Psychologists and dieticians were heavily engaged in the QTs (9 people).

#### Quality of care at the centre

Table 5 presents the items that achieved consensus or dissensus among the patients/parents and the professional groups on items related to Quality of care and organizational features at the centres after three years of joint QI work.

**Table 5:** Consensus and dissensus between the P&P and the Professional groups on Quality of care and Organizational features at the centres

Categories: Quality of care, Patient centredness, Leadership	Consensus amongst P&P	No consensus amongst P&P
Consensus amongst Professionals	Quality of Care:	Quality of Care:
	(++) Existence of improvement goals at the CFC and indicators to monitor them (++) Existence of a therapeutic education program and professionals trained to deliver it (++) Adequate multidisciplinary team, stable over time and possessing expertise in CF care (++) Optimized clinic visit process allowing the patient to see all members of the core team and any referral professionals from various disciplines when necessary (++) Optimized process of answering phone or email messages from patients and families (++) Existence of an electronic patient record system at the centre	(NC,+) Periodic review of the records of the patients who came to the CFC, during the multidisciplinary staff meetings (NC,+) Availability of care guidelines to all professionals (NC,+) Organization of care providers in the patient community
	Patient Centredness:	
	(++) Taking patient needs and requests into account (++) Analyzing causes of complaints to prevent problems from recurring	
	Leadership:	
	(++) Driving the organization to meet patient needs and ensure safety of care	
No consensus amongst Professionals	Quality of Care	Quality of Care:
	(N,NC) Use of the EPR by the team during the staff meetings (N,NC) Existence of a procedure to inform professionals on updates to guidelines	(NC,NC) Patient therapeutic education meeting patients’ needs (NC,NC) Biology or Imaging Information contained in the EPR
		Patient centredness:
		Using data from the patients themselves to improve services

All the items that achieved a strong positive consensus among the patients and parents also achieved a strong positive consensus among the professionals on the QTs. They were related to the following domains of the chronic care model: 1) GOALS: the existence of improvement goals at the CFC and indicators to monitor them, 2) SELF-MANAGEMENT SUPPORT: the existence of a therapeutic education program and professionals trained to deliver it 3) MULTIDISCIPLINARY CARE: an adequate multidisciplinary team, stable over time and possessing expertise in CF care, as well as KEY PROCESSES OF CARE: an optimized clinic visit process allowing the patient to see all members of the core team and any referral professionals from various disciplines when necessary as well as an optimized process of answering telephone or email messages from patients and families 4) INFORMATION SYSTEM: the existence of an electronic patient record (EPR) system at the centre.

Items detailing patient therapeutic education in practice, as well as items regarding certain information contained in the patient record achieved no consensus neither in the patient/parent group nor in the professional group.

The patients and parents granted unanimous neutral response (“Don’t know”) to items regarding the use of the EPR by the team during the staff meetings and the existence of a procedure to inform professionals on updates to guidelines when the professionals showed no consensus on these items.

Three items achieved a strong positive consensus among the professionals only. They were related to the following domains of the chronic care model: 3) MULTIDISCIPLINARY CARE: the systematic review of the records of the patients who came to the CFC; 5) DECISION SUPPORT: the availability of care guidelines to all professionals and 6) COMMUNITY NETWORK: the organization of a network of professionals in the patient community for managing care at home.

#### Organizational features at the centre

Unanimity was achieved for items related to PATIENT CENTREDNESS, taking patient needs and requests into account and analyzing causes of complaints to prevent problems from recurring. However, no consensus was achieved with respect to using data from the patients themselves to improve services*.* The same results were observed for the responses of the professionals with a rate of agreement of more than 90% on the first items, and a lower rate of agreement (< 70%) on using data from the patients themselves.

A consensus was achieved both in the patient/parent and in the professional group in perceiving LEADERSHIP as driving the organization to meet patient needs and ensure safety of care. Other aspects of leadership related to the multidisciplinary team management were mostly answered by patients/parents with “Don’t know”. The responses of the professionals by centres, displayed along the 5 axes of “radar” graphics, also show different types of leadership across the centres.

#### PHARE-M performance and QT effectiveness

Table 6 presents the items that achieved consensus or dissensus among the patients/parents and the professional groups on items related to the program’s performance and the QTs’ effectiveness.

**Table 6:** Consensus and dissensus between the P&P and the Professional groups on PHARE-M perceived performance and QT effectiveness

Categories: PHARE-M performance QT effectiveness	Consensus amongst P&P	No consensus amongst P&P
Consensus amongst Professionals	Experience on the QT:	
	(++) Satisfied with my experience as a member of the QT (++) Wish to remain on a similar team working on QI	
	QI work done by the QT:	
	(++) Usefulness of the work done by the quality team in improving care (++) QI work meets the organization’s needs (++) An ongoing quality improvement process has to be maintained to continuously improve care at the centre	
	Mastery of PHARE-M method and tools:	
	(++) A clear vision of the area to focus the improvement efforts on (++) A guide for organizing the QI work (++) Ability to implement changes (++) Ability to analyze data to ensure changes were improvements (++) Need to set up a specific data collection for QI work	
No consensus amongst Professionals		Mastery of PHARE-M method and tools:
		(NC,NC) Ability of the QT to analyze variations in processes over a period of time (NC,NC) Availability in routine of data to analyze and identify problems (NC,NC) Availability of routine data collection to follow the implementation of the new processes of care
		Change Management (PDSA cycles):
		(NC,NC) Ability to conduct tests of changes with PDSA cycles and learn from the results (NC,NC) Support from the other hospital departments to conduct changes

The perceived performance of the PHARE-M was expressed with items focusing on the experience of the respondents as members of the QTs. A strong positive consensus was achieved amongst both patients/parents and professionals regarding their satisfaction as a member of the QT and their wish to remain on a similar team working on QI. Moreover, their perception of the usefulness of the work of the team in improving care and meeting the organization’s needs was unanimously positive. All stated that an ongoing quality improvement process had to be maintained to continuously improve care at the centre*.*

The performance of PHARE-M as a “training-action” program on this QI approach was appreciated by the respondents with items characterizing their mastery of the quality methods and tools. There was a strong positive consensus in the two groups that the PHARE-M led to a clear vision of the area on which to focus the efforts for improvement at the centre, provided a guide for organizing QI work, and enabled the team to change its way of working and analyze data to ensure that these changes represented an improvement. Both groups agreed that a specific data collection had to be established for the QI work. The others topics related to the availability of data at their centre, by the end of the program, to allow to analyze and identify problems as well as to follow the implementation of changes achieved no consensus neither in the patient/parent group nor in the professional group.

Regarding the techniques to lead changes, no consensus was achieved in both groups on PDSA cycles monitoring to implement changes through tests and evaluations before extension. The support for changes implementation from the other departments in hospital achieved no consensus among the two groups.

#### QT functioning

Table 7 presents the items that achieved consensus or dissensus among the patients/parents and the professional groups on items related to the QT’s functioning. Those items address successively QTs process strategies, decision-making in the QTs, normative management, and internal or external collaborations [21].

**Table 7:** Consensus and dissensus between the P&P and the Professional groups on QT functioning

Categories: QT functioning	Consensus amongst P&P	No consensus amongst P&P
Consensus amongst Professionals	Process strategies:	Process strategies:
	(++) Leader’s behavior reflecting the importance he/she placed on the quality team functioning well (++) Members of the team came from different backgrounds, experiences and skills (++) Availability of enough resources and skills on the team to work properly (++) Team receiving all information required to plan and organize its work	(NC+) The leader also asked the opinions of the other members of the team
		Decision Making:
		(NC+) We appreciated and built with our differences
		Normative:
		(NC+) The team members were all focused on achieving the same goals.
	Decision Making:	
	(++) Attention being paid to the contributions of each member of the team (++) Most team members participating in decision-making (++) Ease for all members in suggesting ideas for change	
	Normative:	
	(++) Team members agreed on the project’s objectives (++) The achievement of the objectives guided the activities of the members of the team.	
	Internal/external collaborations:	
	(++) The people I’ve worked with are comfortable suggesting changes and improvements	
No consensus amongst Professionals		Normative:
		(NC,NC) The team members did what was expected of them. Internal/external collaborations: (NC,NC) There was a lot of cooperation between the departments of the hospital.

A strong positive consensus was achieved on the items describing QT process strategies: the leader’s behavior reflecting the importance he/she placed on the quality team functioning well, the team receiving all information required to plan and organize its work and, the availability of enough resources and skills on the team to work properly. The process of shared decision making on the team was rated as highly positive with attention being paid to the contributions of each member of the team, most team members participating in decision-making, and ease for all members in suggesting ideas for change. The normative regulation on the QTs was rated high regarding the agreement on and achievement of the objectives of the QI project. Though consensus was achieved on the professionals group on all members focusing on achieving the same goals, there was no consensus among the patient/parent group on this item. Last, internal collaborations in the QTs were rated high in the two groups but no consensus was achieved on external cooperations with the other departments of the hospital.

#### Patients and parents involvement in the PHARE-M

Table 8 presents the items that achieved consensus or dissensus among the patients/parents and the professional groups on items related to Patient and Parent Involvement in the PHARE-M.

**Table 8:** Consensus and dissensus between the P&P and the Professional groups on Patient and Parent Involvement

Categories: P&PI	Consensus amongst P&P	No consensus amongst P&P
Consensus amongst Professionals	Activation/Recruitment:	Activation/Recruitment:
	(++) The presence of a patient or parent on the quality team is “a given and an asset” (++) Importance of the information provided to the patient or parent regarding the QI program goals (++) Need for a good relationship between the care team and the patient/parent involved	(NC,+) The patients and parents are informed regularly (annually or more often) by the team about general subjects concerning cystic fibrosis care and research. (NC,+) P&P must have “required qualities” to join the team
		Empowerment:
		(NC,+) P&P have taken a step back and drawn general lessons from their own experience (NC,+) The patient or parent is also motivated to improve his or her own management by participating in the program.
	Empowerment:	
	(++) P&P role on the QT has to be conveyed to the other patients or parents followed up at the centre (++) The patient or parent is motivated to improve care for all (++) The organization of the PHARE-M throughout France created good conditions for their membership on QTs	
	Contribution:	
	(++) The patient or parent participates in and contributes significantly to the work of the QT. (++) Their ideas and proposals were generally taken into account (++) The patient or parent’s regular participation at team meetings at the CFC is indispensable.	
No consensus amongst Professionals	Activation/Recruitment:	Activation/Recruitment:
	(+NC) Patients/parents should have developed copying skills (with the disease)	(NC,NC) The patients and parents are rather familiar with general cystic fibrosis information: research, progress made, and Registry data
	Empowerment:	
	(+NC) Reimbursement of P&P travel fees	Empowerment:
		(NC,NC) The participation of a patient or parent should be facilitated by the reimbursement of other expenses: child-care, lost working hours, etc. (NC,NC) P&P need to be knowledgeable about the disease and its management beyond the requirements of their own care (NC,NC) The participating patient or parent does not represent all patients (NC,NC) It would be necessary to include several patients or parents to ensure that more different points of view are represented (NC,NC) P&P need to understand the general functioning of the hospital
		Contribution:
		(NC,NC) The participation of a patient or parent on the team at French national training and information meetings is indispensable. (NC,NC) The patient or parent participated and contributed as much as the professionals during the French national meetings (NC,NC) The atmosphere of work at the QT meetings is better and more productive when the P&P is present. (NC,NC) The pace of work is slower when the patient or parent is present at the QT meetings. (NC,NC) Certain decisions made by the QT are inspired by the patient/parent.

The first series of items concerned the selection and activation of the patient/parent recruited. There was a consensus that the presence of a patient or parent on the quality team was “a given and an asset” despite a possible lack of education or their personal problems. A strong consensus was found to recruit a patient or parent well informed regarding the QI program goals and the need for a good relationship between the team and the patient/parent involved*.* The development of coping skills (*knowing how to manage emotions and stress; solving problems, making decisions, and making choices; knowing how to communicate and being at ease in relationships with others; and knowing how to put oneself in the place of others)* was by consensus a requirement for the patients and parents to be recruited to the QT. These items also achieved a strong consensus among the professionals, who had a higher rate of agreement on the “required qualities” for the patient or parent to join the team. Those qualities were not explicitly stated in the questionnaire.

Three items achieved a consensus among the patients and parents regarding their empowerment for participation: the reimbursement of their travel fees, their high motivation to improve care for all – achieving a weaker consensus to improve care for themselves, and the fact that their role on the QT was conveyed to the other patients or parents followed up at the centre. Only 8 out of 12 patients/parents agreed on the need to be knowledgeable about the disease and its management beyond the requirements of their own care – while professionals had no consensus on that need. The professionals had a higher rate of agreement on the importance of the patients and parents taking a step back and drawing general lessons from their own experience. No consensus was achieved in both groups on the need for the patient or parent involved to understand the general functioning of the hospital. Finally, the patients and parents unanimously indicated that the organization of the PHARE-M throughout France promoted their membership on QTs.

Regarding their contribution to the QI work, the two groups agreed that patients and parents could make significant contribution to the work of the quality team and that their ideas and proposals were generally taken into account. Both groups agreed that patients and parents had to participate in the local QT meetings – rather than in the national meetings, to make these contributions. No consensus was achieved in both groups on the assertion that certain decisions made by the quality teams were inspired by the patient/parent.

## Discussion

Following the results of the investigations conducted with the care providers and patients/parents, we review the highlights on the instrumentality of the method to involve patients and parents in PHARE-M QIP. We then discuss the initial questions raised about this partnership during the PHARE-M program in France and propose a list of success factors which seem essential to long term patient/parent involvement in QI work in Table 9.

**Table 9:** Success factors sustaining long term patient and parent involvement in QI projects

Factors related to patients and parents:
• Good relationship with the care team • Coping with the disease, its complications and the effects of treatments • Stable health condition of the patient or the child of the parent • Stable socio economical family situation • Motivation to improve care for all (beyond improving care for oneself) • Possibility of involving more than one patient or parent in the team to insure the presence of one of them at each meeting and to bring diverse experiences to the discussions (for instance parents of children of various ranges of age or transplanted and non transplanted patients…) • Ability to give time to the project, participating to the trainings and local meetings, and availability of communication tools (internet) at home
Factors related to the care team:
• Mature relationship with the patient/parent: readiness to a partnership for care, being at ease with shared decision making and/or patient education • Leadership wishing to involve patients/parents on a long-term basis, « playing the rule » of transparency and effectively taking the responsibility for the project and for the change actions implemented • One professional being the correspondent of the patient/parent for the QI project solving practical issues • Awareness to the guidelines and consensus for care and ability to discuss/share them with the patient/parent • Attention paid to psychosocial difficulties encountered by the patient potentially contradictory with their involvement
Factors related to the QI method
• Present the involvement of a patient/parent as a pre-requisite to engage in QI work, based on literature and a « safe » framework to recruit them • Take the financial charge of patient and parent involvement at the program level (thanks to an agreement with the patient organizations if possible) • Offer an appropriate set of communication tools towards the patients/parents followed at the center, including the patient group if any, as well as towards the hospital administration • Provide the same training on the quality methods and tools to the professionals and the patients/parents involved • Install resources for the QI work at the centre and manage the regular participation of the patient/parent or his update on the project • Secure the framework with ethical rules allowing full participation of all members, recalling roles and responsibilities • Offer new perspectives to the whole teams including the patient or parent involved, facilitating benchmarking with other practices, • Provide access to guidelines and consensus for care to the whole team including to the patient or parent • Provide an on-site Coaching to support the team in analyzing their processes of care from the point of view of the patient/parent (shadowing a patient) and reinsuring the place of the patient/parent involved • Consider that the results achieved are attributable to the whole quality team and beyond, to the multidisciplinary team who implement the new process of care, and not to one member in particular, be it a patient/parent or a professional

### Highlights on the method to involve patients and parents in PHARE-M

PHARE-M quality improvement program was innovative in France in 2012 as it intends to install a culture of quality improvement in the CF care teams, focusing on patient outcomes improvement and process of care redesign. Patients and parents were involved on a long time period with the care teams at their centre to work together on quality improvement of care.

- Conditions for patient and parent recruitment

The essential selection criteria underlined by both patients/parents and professionals were a good relationship with the team, a desire to improve care for all patients and a willingness to take a step back and draw general lessons from their experience with the disease. Training on the general functioning of the hospital or the management of the disease have not been offered at recruitment and didn’t appear to be a pre-requisite for participating. The professionals contributed their in-depth knowledge of the disease and its treatments to the discussions. This was made easier by the stability, expertise and experience of the team members. Extensive information on the program provided to the other patients or parents of patients followed up at the centre as well as to the hospital administration was indispensable to legitimize the participation of the patients and parents. Nevertheless, three parents stopped their participation at the end of the first year for reasons related either to the physician at the centre or to a worsening in the patient’s health status. This illustrates the impact of the medical leadership on patients/parents’ long-term involvement and confirms that a stable health condition on the part of the patient is often necessary to engage or stay in such a program [4].

- Participation at the quality improvement national training meetings

The participation of patients/parents in the national training meetings about the QI method and tools was an integral part of the program. The reimbursement of their travel fees appeared to be mandatory to enable them to participate at these training meetings. Such participation gave all team members an equal opportunity to be trained in the quality improvement method. Given that none of the « students » had any prior knowledge of this particular quality approach, despite their different professional expertise and background, they all engaged in discussions effectively. The transparency of the outcomes from all centres involved at these meetings was another aspect of the method [12]. It provided results from the patient registry report by centre comparing patient health outcomes to identify potential best practices at some centres. Although this transparency was novel within the French CF care network, it was well accepted by the professionals and well received by the patients and parents, as it led to the choice of a theme for improvement at the centre. Condition for effective partnership between professionals and patients in QI work involved transparency of the results and the commitment to improve them [10]. Given that the goals were clear and shared from that time forward, the patients, parents and professionals were equally committed to achieving them during the program [25]. Moreover, the collaborative aspect of the program created a community of centres willing to continue sharing their work on quality improvement and their results as part of an open process of « benchmarking of practices » [11].

- Contributions made by patients and parents

The contributions made by patients and parents obviously depended on their frequent participation in the QT meetings at their centre. The experience of the patients and parents was brought to the discussions using questionnaires during the clinic visits or phone calls as well as patient shadowing during clinic visits and observation of multidisciplinary staff meetings. The joint work on these processes resulted after three years in the shared opinion of having implemented optimized processes. The patients and parents sometimes also contributed their own expertise (quality, IT, communication etc.…) by « specific tasks » assigned to them depending on their wishes, availability and own expertise. Some examples were cited in the comments: a multi-purpose notebook was created to communicate with the care team about events at home, treatments prescribed and educational material; internet surveys were developed and the results were analyzed for the QT; a dashboard of indicators in the form of a smiley face was develop for the children to assess their care at the end of the visit; a « gazette » about the QI program was issued by parents and adolescents; a bulletin board was created to display information about the QI project in the CFC. These contributions seem to have accelerated the QI work of the team and facilitated communication with the other parents/patients. Most often, it was ultimately difficult to attribute certain changes in the centre organization and process of care specifically to any specific team member – patient, parent or professional.

### Questions raised by this partnership during PHARE-M in France

The following questions were raised by the stakeholders of the PHARE-M program, including the professionals’ and the patients/parents’ representatives, on the feasibility, efficiency and utility of this partnership during the program.

- How were perceived the conditions in place to allow the participation of patients and parents in the program?

The patients/parents as well as the professionals agreed that the organization of the PHARE-M throughout France created good conditions for their membership on QTs. All the respondents were satisfied with their experience, mostly favorable to further participation on a similar quality team and agreed with the necessity of an ongoing quality improvement process to continuously improve care at the centre. These opinions reinforce the French national PHARE-M team’s belief that the program enhances the involvement of patients/parents along with their care teams to improve care at their centre. It also indicates that the participation in the program does not cause deleterious effects to the patients/parents involved, which could have come from the vision of the “defects” seen in the management of care at their centre.

Some items remained not consensual: they may be addressed through further experimentations during the next sessions of the program. They concern “the participation of a patient/parent should be facilitated by the reimbursement of other expenses such as child-care, lost working hours...”; “the necessity to include several patients or parents to ensure that more points of view are represented” and, “the need for patients/parents to understand the general functioning of the hospital”. At the beginning of the program, questions about « representativeness » of the patients/parents involved were evoked. Should those involved be individuals recruited by the care teams according to the mentioned criteria or national patient organization or local patient group representatives, when they exist? Is the experience of patients/parents involved sufficient to inform QI work? Should the experience of other patients and parents be captured to complement their own? These questions raise matters of legitimacy, democracy and responsibility. In the frame of our QI project, the legitimacy of the patient and parent involved appeared to be granted by the care team and not by a patient organization or patient group. It happened in some settings that the parent was a member of the CF local patient group but their involvement was decided upon by the care team and not requested by the patient group. Their position in the quality team did not change the rules for communication between the quality team and the patient group. It was clear that the patient or parent involved spoke to their own experience and not to that of a group of patients/parents. These questions are important and should be clarified at the meso- and macro-system level to facilitate and foster patient involvement in the quality improvement work with their care team, as it has been done for patient representation in hospital committees. Financial aspects related to the participation of the patient/parent in meetings with the care team, in particular travel fees or other allowances, could be part of this clarification.

- How did the quality team’s professionals perceive this participation and what were the feelings of the participating patients and parents?

At the introduction of the program, barriers from professionals as well as from patients and parents had to be overcome. In the interviews, the switching of roles in parents (I come as a parent to the consultation, and in the quality group I commit myself as a user/ a designer of the process) and in patients (I come as a patient to the consultation, and I commit myself in the quality group as a user/improver) creates a tension between those positions of the patients/parents. The potential for tension arose when they didn’t feel satisfied with their experience of the care delivered by the team or with the quality of communication with certain members of the team, and when they had not coped with a previous painful circumstance such as the diagnosis of CF for their child or the management of a complication of the disease. The attenuation of this tension is critical to gradually increase the involvement of parents and patients during the QIP. This attenuation was observed in the results of the investigations after three years, which lets us hypothetize that the QIP might have acted as a process of resilience for patients, parents and professionals.

A shift in the representation of care by professionals and patients/parents was observed in the course of the program towards a co-produced service which co-production is based on a mutual understanding of roles and competences, mutual participation in communication and actions and respective responsibilities in delivering care. French teams that had previously developed a culture of patient therapeutic education and were used to partnering with patients/parents for their own care, were more favorable to patient and parent involvement in care QI work than the teams that had not. This observation, and whether the other teams have overcome their initial reluctance, will have to be further analyzed in the results by centre, as there was a high consensus after three years that “the presence of a patient or parent on the quality team is a given and an asset”. Our experience confirms that the more the professionals and the patients collaborated to plan and develop services, the more this collaboration was accepted among both the professionals and the patients [14].

Upstream conditions could be created to support the participation of patients/parents in the health system, especially in quality of care improvement programs along with their care team. In Canada, a framework for interprofessional education and collaborative practice was developed to address the needs in terms of skills and behaviors for professionals engaged in collaborative practice with healthcare practitioners, patients, families and communities [26]. Six domains were identified: interprofessional communication; patient and family centered care; role clarification; team functioning; collaborative leadership; and interprofessional conflict resolution. Several assumptions underpin this framework one of them being that interprofessional practice is not innate but requires a consistent culture of learning and practice. Further reflection would be needed to refine such a framework to the French system of health continuing education and thus foster the necessary shift towards patient involvement in quality of care improvement programs [27].

- Does the patient or parent have a different vision than the professionals on the quality of care achieved after three years of joint work?

After three years of joint work, the awareness of the patients and parents on care organization and processes at their centre was high – similar to that of the professionals – concerning matters relevant to them: multidisciplinary care, patient education, the clinic visit process… But their awareness on some aspects of the organization such as the information system (patient electronic record) and the management of care guidelines, remained low. Even so, these aspects are not to remain fatally out of their attention for quality of care improvement: the impact of educating parent in care guidelines on clinician adhering to them has been demonstrated in a pediatric CF program [28] and patient-led training in medical education has had an impact on the application of safety guidelines by clinical teams [29]. In Sweden, patient electronic records have been opened to allow patients access to their health record and provide input such as the schedule of the next visit, results on health outcomes followed at home and various mailings [30]. When these matters are explicitly shared with them as part of their care, patients and parents will probably be able to contribute to improve these fields by reporting their experience and needs.

- Was the participation of patients and parents in the discussions and in decision making with the other team members effective?

All members felt that they could participate in decision-making, that attention was paid to their contributions and were at ease in suggesting ideas for change. The goals were clear and shared, which probably channelled the discussions amongst the members of the QTs who came from different backgrounds, experiences and skills. The patients / parents’ contribution was highly appreciated but changes in the organization or process of care were not specifically attributable to them.

## Conclusions

### Reflections for further experimentations and research on involving patients’ views in quality of care improvement programs

Our experience of patient/parent involvement in the PHARE-M QIP raise matters in relation to the nature and extent of the patient experience incorporated in the QI work. In 2005, Bate et al. defined the concept of experience-based design (EBD) as a new way of co-designing health services with the patient in a context where they are no longer a « passive recipient of a product or service » but are « integral to the improvement and innovation process » [31]. Like other design sciences – such as architecture, healthcare is associated with the three aspects of functionality (*how well it does the job and fit its purpose - performance*), safety (*how safe and reliable it is - engineering*) and usability (*how the user interaction with the product or service is experienced*). According to Bate, *EBD is a user-focused design process with the goal of making user experience accessible to the designers, to allow them to conceive of designing experiences rather than designing services*. Which consequences such a vision has on QI work in healthcare? First, patients are incorporated for their experience of care, not necessarily for any prior expertise they may offer. Second, words are used to translate events (adverse or positive events) into experiences which may then be presented in the form of storytelling, sometimes played by actors. Third, experience amounts to more than views, complaints or satisfaction; it features *almost everything that is required to understand strengths and weaknesses and what needs to be redesigned in the care process*. For all these reasons, the acquisition and use of patient experiences in care improvement is a specialized activity which needs to be learned and practiced. It represents one valuable way to incorporate the patient experiences into care improvement [32].

To address the question of patients’ experience incorporated into QI work, specific « patient experience surveys » have been drawn up in some countries [33, 34]. These surveys intend to collect information on the care pathway and on the characteristics of the care delivered to the patient in the previous months. They are designed to reflect the care that the patient should have received according to the standards of care for the disease. If they are administrated in ways that insure a good response rate from patients and parents, they enable the preparation of a center report of Patient Reported Outcomes in terms of quality of care [35]. They may provide information about the variability of care across geographic or socioeconomic dimensions and avenues for quality of care improvement. These instruments help fill the gap between individual experiences of care and the general features of the care delivered to most patients.

We cannot conclude without comparing the commitment of patients and parents who accept or sometimes claim to be involved in QI programs to the activism defined by Rabeharisoa [36]. This commitment actually takes up the main features characterizing patient activism:

1. Include and shape the experiential knowledge of patients and parents;
2. Articulate it with credential knowledge in clinical, organizational and quality fields;
3. Reframe what is at stake, that is co-redesign the process of care;
4. Defend the cause: “the best possible care here and now for all patients”; and
5. Organize a network of expertise with credentialed experts in quality, patient therapeutic education, and academic instances.

### Limitations of the study

Our research has some limitations. First, the sample of centres as well as patients/parents, all of which volunteered to engage in the PHARE-M QIP sessions and test the program before its roll-out throughout France, may not reflect general opinion at all CF centres in France from 2011 to 2015. Second, the appearance of numerous publications and mediated interventions in favor of taking patients’ voices into account in healthcare services has triggered a beginning of a cultural shift in the last years in France. A movement called « Démocratie en Santé » emerged in France in 2015 building on this trend. In the latest PHARE-M sessions, it becomes more obvious to professionals as well as to patients and parents that the latter should be systematically involved in the QI work at the centre, and sometimes more than one at a centre. Their recruitment becomes also easier. It is hoped that arrangements will be made to facilitate patient participation in quality improvement of care, which will in turn have to be evaluated.
